# A Multicenter Study by the DENO Research Group on the Use of Denosumab in Giant-Cell Tumors of the Bone

**DOI:** 10.3390/jcm14093242

**Published:** 2025-05-07

**Authors:** DENO Research Group, Carolina de la Calva, Manuel Angulo, Paula González-Rojo, Ana Peiró, Pau Machado, Juan Luis Cebrián, Roberto García-Maroto, Antonio Valcárcel, Pablo Puertas, Gregorio Valero-Cifuentes, Óscar Pablos, Miriam Maireles, María Luisa Fontalva, Iván Chaves, Aida Orce, Luis Coll-Mesa, Israel Pérez, Fausto González, María del Carmen Sanz, Isidro Gracia

**Affiliations:** 1 Hospital Universitario y Politécnico La Fe, 46026 Valencia, Spain; mangulosa@hotmail.com (M.A.); paulagonzalezrojo@gmail.com (P.G.-R.); 2 Hospital de la Santa Creu i Sant Pau, 08025 Barcelona, Spain; apeiro@santpau.cat (A.P.); pmachado@santpau.cat (P.M.); igracia@santpau.cat (I.G.); 3 Hospital Clínico San Carlos, 28040 Madrid, Spain; juanluiscebrian@gmail.com (J.L.C.); marotocot@gmail.com (R.G.-M.); 4 Hospital Clínico Universitario Virgen de la Arrixaca, 30120 Murcia, Spain; avd1981@hotmail.com (A.V.); jppuertasgsandoval@gmail.com (P.P.); gregorio.valero29@gmail.com (G.V.-C.); 5 Hospital Universitario de Bellvitge, 08907 Barcelona, Spain; opablos@bellvitgehospital.cat (Ó.P.); miriammairelesperez@gmail.com (M.M.); mfontalva@bellvitgehospital.cat (M.L.F.); 6 Hospital Universitario Nuestra Señora de Candelaria, 38010 Santa Cruz de Tenerife, Spain; ivanchd92@gmail.com (I.C.); aidaorce@gmail.com (A.O.); lcolmes@gmail.com (L.C.-M.); 7 Hospital Universitario Ramón y Cajal, 28034 Madrid, Spain; israel.perezmun@salud.madrid.org (I.P.); faustogonzalezlizan@gmail.com (F.G.); carmensanzp@hotmail.com (M.d.C.S.)

**Keywords:** giant-cell tumors of the bone, denosumab, neoadjuvant, adjuvant, single treatment

## Abstract

**Background/Objectives**: Despite the therapeutic potential of denosumab for the treatment of giant-cell tumors of the bone (GCTBs), there is a lack of standardization in treatment protocols. **Methods**: We present a multicenter, retrospective, descriptive study conducted across the seven hospitals in Spain affiliated with the DENO Research Group. Seventy-three patients diagnosed with GCTB and treated with denosumab were included and stratified according to treatment strategy—neoadjuvant (*n* = 38), adjuvant (*n* = 8), and single treatment (*n* = 27). **Results**: Patients in the neoadjuvant group received denosumab for a median of 6.1 months, with reintroduction after surgery in 25.8% of all cases. Among the neoadjuvant patients treated with curettage, recurrence was 35.5%, with no association with denosumab treatment duration (*p* = 0.274) nor with denosumab reintroduction after surgery (*p* = 0.405). In the adjuvant group, those who completed treatment received denosumab for 15.3 months, while those still undergoing therapy received it for a median of 12.8 months; only one case (12.5%) recurred. Recurrence rates in neoadjuvant and adjuvant treatment strategies were not different (*p* = 0.394). Patients treated only with denosumab and no longer on treatment had received it for 34.2 months, with 31.3% recurrence; those still on treatment had received it for 51.8 months, with 25.0% recurrence. Across all strategies, more than 85% of patients reported favorable clinical outcomes, and only 43.8% presented adverse events. No deaths occurred during this study. **Conclusions**: Although patients who experienced recurrence during neoadjuvant treatment had longer durations of denosumab administration, the difference was not statistically significant. Similarly, recurrence rates did not differ significantly, whether denosumab was reintroduced after surgery or not. Among the patients treated with curettage, recurrence rates were comparable between neoadjuvant and adjuvant strategies. Discontinuation of the single treatment did not necessarily result in disease progression.

## 1. Introduction

Giant-cell tumors of the bone (GCTBs) are uncommon neoplasms mainly affecting skeletally immature young adults. Although they often present as a lytic lesion in the meta-epiphysis of long bones, they may also occur in areas such as the sacrum or the pelvis, which are less amenable to surgical treatment, given their proximity to critical neurovascular areas. In up to 29% of cases, these tumors may extend to the surrounding soft tissues. Despite their low tendency to metastasize, GCTBs can be locally aggressive, causing bone destruction, pain, and morbidity, which usually negatively impact patients’ quality of life [1,2,3,4].

Historically, the mainstay of GCTB treatment has been surgical resection, with an emphasis on preserving as much soft tissue as possible, particularly around articular surfaces, so as to not limit the patient’s function. While intralesional curettage minimizes bone resection, local recurrence rates as high as 50% have been reported for the procedure. On the other hand, although resections en bloc are associated with lower recurrence rates, they tend to result in higher mortality and other related risks, which means that they are typically reserved for the most severe cases [4,5,6].

The advent a decade ago of denosumab, a human monoclonal antibody with high affinity for RANKL (receptor activator of nuclear factor kappa-B ligand) resulted in a significant paradigm shift in the treatment of GCTBs. Moreover, denosumab has, in the last few years, been used in various capacities—as a neoadjuvant agent to facilitate surgery in the event of difficult-to-access tumors or to minimize the aggressiveness of surgery; as an adjuvant agent to reduce postoperative recurrence rates; or even as single therapy in unresectable tumors [1,7,8,9].

Despite all the advances made, the substantial heterogeneity in the literature regarding locations and therapeutic strategies has made it impossible to agree on an optimal treatment protocol [7,10,11,12]. The present multicenter retrospective descriptive study aims to shed some fresh light on the clinical realities of GCTB treatment with denosumab in Spain.

## 2. Materials and Methods

This is a multicenter, retrospective, descriptive study on the treatment of GCTB with denosumab conducted by the DENO Research Group within the framework of a program sponsored by the Spanish Musculoskeletal Tumors Research Consortium (LIETAL) and the Spanish Society of Orthopedics and Trauma Surgery (SECOT). Approval was obtained from the Ethics Committee of the La Fe University Hospital (Valencia) and by the Spanish Medicines Agency (AEMPS) (Approval code JFT-DEN-2019-01). After being informed about the nature of the study, all the patients gave their informed written consent to participate.

As per the inclusion criteria, only patients with a pathological diagnosis of GCTB could be enrolled in this study. All patients had been treated between 2009 and 2019 in one of seven Spanish hospitals benefitting from a sarcoma and musculoskeletal tumor board. All of them had been followed up for at least 6 months and had received the standard denosumab schedule recommended by the manufacturer of 120 mg administered subcutaneously once a month, with two extra doses on the eighth and fifteenth day during the first month. Calcium (2500 mg) and vitamin D (>400 IU) supplements were also administered to prevent hypocalcemia. Patients with a history of conditions related to the calcium and phosphate metabolism, as well as pregnant women and subjects already treated with denosumab, were excluded from this study.

Patients were treated following one of the following three strategies. Subjects with locally advanced GCTB, where only a highly aggressive surgical approach was viable, were treated with a neoadjuvant strategy, with denosumab being administered as initial treatment and followed by a surgical procedure that was not overly aggressive. Patients who underwent surgery and were considered at high risk for local recurrence—due to uncertainty regarding the complete excision of tumoral tissue—received adjuvant therapy. Lastly, single treatment with denosumab was reserved for cases deemed unresectable, where surgical intervention would entail substantial morbidity and/or significant functional impairment due to the tumor’s anatomical location.

All patient demographic and clinical data (sex, age, origin, location, and stage of the tumor according to Campanacci’s radiological grading system [13]) were recorded. A record was also made of the date of initiation of denosumab, as well as of the date of discontinuation of the drug in patients where administration was stopped. In the absence of standardized protocols, therapeutic rest periods were implemented only in cases where the medical team considered that both clinically and radiologically favorable responses sustained over time justified them. In patients undergoing surgery, the type of surgery performed, the kind of local adjuvants administered, and the type of reconstruction carried out were all documented.

During follow-up, an analysis of the patients’ clinical and radiological response was performed. The clinical response was considered favorable when patients reported a decrease in pain in the tumor area for more than 14 days and unfavorable if they reported an increase in pain during the same period. The radiological response was evaluated following the Inverse Choi Density/Size (ICDS) criteria [14]. For practical purposes, only the data obtained at the assessment carried out on completion of the study are shown for patients exhibiting varying responses during follow-up. All the recurrences observed over the course of the study were duly documented, as was their local or systemic nature.

A record was made of all adverse events, verifying whether they coincided with those associated with denosumab in the published literature. Their level of toxicity was established following the Common Terminology Criteria for Adverse Events (CTCAE—Version 5.0) [15].

All statistical analyses were conducted using Stata Statistical Software (16.1, StataCorp. 2019. StataCorp LLC., College Station, TX, USA). A statistical description was made of the data, which are presented as mean ± standard deviation (SD), median (range), or *n* (percentage). The normality of the samples was tested by means of the Shapiro–Wilk tests, and between-group comparisons were performed using Student’s *t* test and Fisher’s Exact Test, as appropriate. To maintain interpretative consistency, recurrence analysis in the neoadjuvant and adjuvant cohorts was limited to patients treated with curettage, as the clinical implications of recurrence differ substantially in cases managed with wide resection or amputation. Cohen’s d statistic was calculated to estimate the effect size of the comparative analyses for continuous variables [16]. Statistical significance was set at a *p* value < 0.05 in all comparative analyses.

## 3. Results

**Figure 1:**
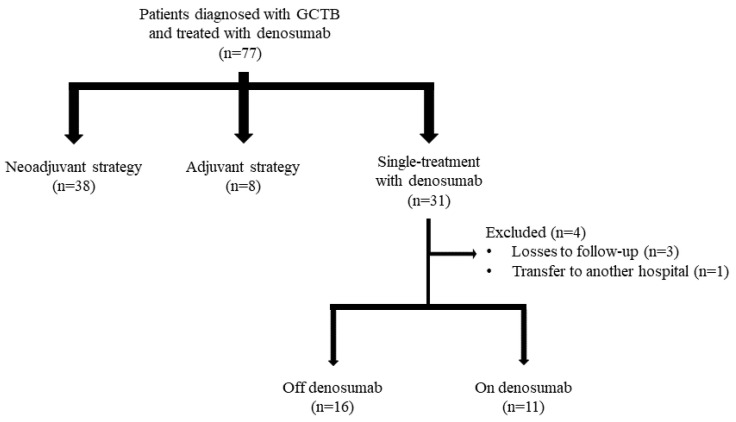
Patient selection process.

Of the initial sample of 77 patients diagnosed with GCTB and treated with denosumab, 4 were eventually excluded (Figure 1). Of the remaining 73 subjects, 38 (52.1%) were treated with a neoadjuvant strategy, 8 (11.0%) were treated with an adjuvant strategy, and 27 (3.0%) received denosumab as a single treatment.

At the beginning of the study, all the patients presented with localized tumors with no systemic disease. Baseline demographic and clinical characteristics are summarized in Table 1. Denosumab administration times stratified by treatment strategy are presented in Table 2. Median follow-up for the whole sample was 52.4 months (6.5–193.4 months). At the end of the study, no deaths occurred.

### 3.1. Neoadjuvant Treatment

Most patients in the neoadjuvant group (86.8%) experienced a clinical improvement, reflected in an 84.2% rate of (total or partial) radiological responses and stabilization of the disease. Median time to surgery after completion of denosumab treatment was 0.7 months (0–3.2 months).

As regards the type of procedure performed, 86.1% of patients were treated with curettage, 11.1% were treated with resection followed by prosthetic reconstruction, and 2.8% had their limb amputated. In 76.3% of cases, the adjuvant method employed was high-speed burring, with liquid nitrogen, ethanol, and phenol being used in 28.9%, 2.6%, and 21.1% of cases, respectively. Reconstructions were carried out mainly with allografts (36.8%) and cement (31.6%). Demineralized bone matrix and tricalcium phosphate were each applied in 2.6% of cases.

Considering only the patients intervened with curettages, in 25.8% of them treatment with denosumab was continued following the procedure. One such patient, who had been started on the drug 6.3 months before completion of the study, was still receiving treatment at the time the study ended. The rest had completed their treatment by the end of the study (Table 2).

Eleven patients (35.5%) on neoadjuvant treatment experienced a local recurrence following surgery. These patients had received denosumab for a longer period (8.3 months) than those who did not relapse (5.7 months), although no statistical differences were found (*p* = 0.274). Nonetheless, Cohen’s d was 0.696, indicating a medium effect size for the duration of denosumab administration in relation to recurrence.

Of the patients for whom denosumab was reintroduced following surgery, 50.0% suffered a recurrence, whereas only 30.4% of those for whom denosumab was not reintroduced exhibited progression of the tumor. No statistically significant differences were found, nonetheless, between both groups in terms of their recurrence rates (*p* = 0.405).

### 3.2. Adjuvant Treatment

All patients on adjuvant treatment were subjected to curettage followed by high-speed burring (87.5%) and phenol application (62.5%) as adjuvant strategies. Reconstructions were carried out using allografts (50.0%), autologous grafts (12.5%), cement (12.5%), demineralized bone matrix (25.0%), and tricalcium phosphate (12.5%).

Postoperatively, patients were started on denosumab within a median of 2.5 months (0–6.1 months). At the last follow-up review, five patients (62.5%) had discontinued denosumab. Four of them had stopped taking the drug, as they were allowed a therapeutic rest period on account of their favorable evolution, while the fifth patient was the only one who had experienced a recurrence, which prompted discontinuation of denosumab and performance of a new surgery. Three patients (37.5%) were still on the drug at the end of the study.

Except for one case where the evaluation was inconclusive, all patients experienced clinical improvement following the combination of surgery and denosumab, with 62.5% of them also demonstrating a complete (or at least partial) radiological response.

### 3.3. Recurrence in Curettage: Neoadjuvant vs. Adjuvant Treatment

Considering only patients treated with curettage, no statistically significant differences were found (*p* = 0.394) between the recurrence rates of those who received denosumab before surgery (neoadjuvant treatment, 35.5%) and those who received it afterward (adjuvant strategy, 12.5%).

### 3.4. Single Treatment

A total of 85.2% of patients treated exclusively with denosumab experienced clinical improvement, reflected in a 77.8% rate of (complete or partial) radiological responses and stabilization of the disease.

Of the 27 patients treated only with denosumab, 59.3% had completed their treatment at the last follow-up review, while 40.7% were still receiving the drug.

Five of the patients who had stopped taking denosumab (31.3%) suffered tumor progression after being treated for a median of 45.5 months (14.0–75.8 months). In one case, such progression was multifocal. In turn, two of the patients who were still on denosumab (25.0%) also experienced tumor progression after being treated for 25.7 and 37.7 months, respectively.

### 3.5. Toxicity

A total of 43.8% of patients presented with some kind of adverse reaction associated with denosumab (Table 3), without any correlation being found between these adverse events and the treatment strategy employed (*p* = 0.230). Median time of administration of denosumab at the time of developing an adverse reaction was 12.7 months, with wide variations between subjects (0.2–92.5 months).

Most adverse events did not result in severe clinical symptoms, although four cases were classified as grade III. One patient receiving neoadjuvant treatment sustained an atypical grade IV femoral fracture, which occurred 11.8 months after denosumab was reintroduced and after a total of 27.6 months of cumulative exposure since treatment initiation. Five patients developed jaw osteonecrosis; four cases were grade II, and one was grade III, all occurring in patients treated exclusively with denosumab. The median time to onset was 50.0 months (3.2–91.0 months). Only 10.7% of patients who discontinued denosumab did so because of toxicity resulting from the drug. No instances of the known adverse events associated with denosumab, such as hypophosphatemia, dyspnea, coughing, or eczema, were reported.

## 4. Discussion

### 4.1. Treatment of GCTB with Denosumab

Although treatment of GCTB with denosumab gained popularity over a decade ago, no standardized administration protocols have been published as of yet. Although most patients are evaluated by multidisciplinary medical boards, therapeutic strategies tend to vary considerably, given the paucity of clinical trials, the low incidence of the condition, and the varying ways in which the drug is used [17,18]. This study presents a descriptive analysis of a series of GCTBs treated in Spanish hospitals using denosumab across three different therapeutic strategies.

The management of GCTB with denosumab remains a matter of controversy. Some authors have suggested that the drug only achieves favorable results temporarily, i.e., as long as it is administered, and that discontinuation of treatment may result in the tumor extending to the surrounding soft tissues. This is possibly one of the reasons behind the variability observed in the duration of administration of the drug, which remains one of the main points to be elucidated before optimal treatment protocols can be established [1]. Moreover, the length of administration in certain studies is influenced by socioeconomic factors. This is typically the case in studies conducted in countries where patients must pay for their treatments out of their own pockets, which means that some of them can only afford fewer doses than those recommended by their medical team [19,20].

On the other hand, however, the clinical benefits of treatment with denosumab, regardless of the strategy employed, appear to be widely recognized. Indeed, most patients experience a rapid improvement in their quality of life due to the lessening of pain. Over 85% of the patients in the present study exhibited a favorable clinical response, which is in line with the results reported in the literature [18,21].

### 4.2. Neoadjuvant Treatment

Treatment with denosumab is chiefly indicated in tumors initially considered inoperable or in those for whom resection would require an extensive excision or even amputation of the limb. The goal is to reduce the size of the tumor, thus facilitating the surgical procedure [18]. The effectiveness of denosumab in this context is well documented in the literature, with several studies reporting that it not only makes surgery possible but also reduces its aggressiveness, simplifying the procedure as a whole. Shorter surgical times have also been reported, as well as a decrease in intraoperative blood loss, as a result of a reduction in the tumor’s vascular density [21,22,23].

Despite the foregoing, neoadjuvant use of denosumab is not exempt from controversy, with some studies suggesting that local recurrence rates, particularly following curettage, could be higher than those for patients not treated prior to surgery [21]. Some authors have theorized that, given that the calcified rim resulting from treatment with denosumab could, for reasons that remain unknown, contain neoplastic cells, curettage is typically unable to fully remove the tumor tissue by itself [17,18]. Thirty-five percent of the subjects in our study who received denosumab as neoadjuvant treatment experienced a local recurrence after curettage. Interestingly, these patients had received denosumab for considerably longer than those in whom the GCTB did not recur. While no statistical significance was reached, Cohen’s d indicated a medium size effect. This, combined with previous findings and our own observations, leads us to believe that the lack of statistical significance may be attributed to the limited sample size, which could have hindered the statistical power.

A review of the literature reveals that patients experiencing the highest recurrence rates are typically those who receive denosumab for long periods of time, usually exceeding 6 months [19,22,24]. In our study, those who experienced recurrence had a mean treatment duration of 8 months, while those who did not experience recurrence had a mean administration time of less than 6 months, consistent with the aforementioned findings. On the other hand, Huang et al. (2023) [20] reported recurrence rates around 20% among patients receiving denosumab for 3 months prior to surgery, without statistically significant differences with respect to the corresponding control groups.

In view of the above, it would seem reasonable to concur with authors who claim that the duration of neoadjuvant administration of denosumab ought to be reduced once an appropriate response has been achieved that allows for performance of the surgical procedure [18,19,21,25]. Although a certain consensus seems to exist in favor of neoadjuvant administration of denosumab for 3 to 4 months prior to curettage and for up to 6 months before more aggressive resections [1,21], certain ultra-short 21-day protocols have also been proposed [26]. We consider it essential for future research to focus on establishing a length of administration of denosumab that can reduce the aggressivity of surgery without increasing the local recurrence risk.

Certain authors have suggested that the recurrence rate following neoadjuvant strategies could be reduced by reintroducing denosumab following surgery. This approach has not been proven effective in all patients, with the literature reporting local recurrence rates between 34 and 60% [17,25,27]. At 50%, the local recurrence rate for patients where denosumab was reintroduced in this study fell within that same range. Although this rate was higher than that for patients in whom the drug was not reintroduced, no statistically significant differences were observed in the recurrence of both groups—neither in the overall neoadjuvant series nor when limiting the analysis to patients treated with curettage. There is a possibility, nevertheless, that conducting an individualized analysis of the consequences of reintroducing denosumab could have resulted in higher recurrence rates for these patients.

### 4.3. Adjuvant Treatment

As far as the postoperative use of denosumab is concerned, we decided to distinguish between reintroductions of neoadjuvant treatment and purely adjuvant treatments in patients who had not received the drug preoperatively. Although there is limited information on the results of these treatment modalities, particularly on the administration of a purely adjuvant treatment to denosumab-naïve patients, there seems to be a trend in both cases towards applying 6-month-long postoperative cycles [25,28]. In the present study, denosumab was reintroduced in a group of selected patients over a median period of 5 months, in line with the 3–24 months range reported in the literature. It must be noted that this period is shorter than the duration of preoperative treatment with the drug [27,29,30]. It should also be underscored that adjuvant treatments in the present study were also longer lasting than those reported in the literature, with a median duration of 12.8 in patients who had completed their treatment and 15.3 months in those still being treated, as compared with 6 and 12 months, respectively [28,31].

Only one of the patients (12.5%) in the adjuvant group experienced a local recurrence. In a recent study, Akyildiz et al. (2024) [31] analyzed 18 patients and found denosumab to be effective and safe as an adjuvant agent, without observing any recurrence. When comparing the recurrence rates of our patients treated with the same procedure—curettage—and the rates of those who received denosumab either as a neoadjuvant or adjuvant therapy, we found no differences associated with the timing of administration. However, we cannot rule out the possibility that these results may have been affected by the small sample sizes.

The paucity of studies on the subject and the generalized lack of awareness of the possibility of using denosumab in the adjuvant setting prevent us from contextualizing our results. Nonetheless, we must reaffirm our belief that the positive results obtained for denosumab in the present study should, for the most part, be attributed to the surgical procedure, with denosumab acting, at best, as a control agent. In the absence of clinical trials looking into the subject, it would appear that adjuvancy with denosumab should only be indicated when the individual patient’s characteristics make it advisable.

### 4.4. Single-Treatment

As regards the duration of single treatment with denosumab, some authors have suggested keeping patients on the drug for life in order to ensure proper tumor control. In the present study, patients on single therapy were treated for a period ranging between 5 and 130 months. This wide variability was influenced by factors such as recurrence during treatment, toxicity, and the implementation of therapeutic breaks following favorable outcomes. Other authors also found similar disparities, for example in cases of unresectable tumors treated only with denosumab, where the drug was used over periods ranging from 4 to 54 months [11,12,25,32,33,34,35,36].

Although the patients in this study were examined in a previous publication by the authors, that previous analysis looked at subjects treated only with denosumab where the drug had been discontinued, stratifying them into those who experienced tumor progression and those who did not [37]. The present study, in contrast, presents results in a more general way, without distinguishing between any particular subgroups. Only 31% of patients who had already discontinued treatment recurred, which is in line with the 15–34% recurrence rates reported in the literature. This suggests that discontinuation of the drug does not necessarily result in a recurrence [11,12,22]. Moreover, denosumab was not able to prevent recurrence in 25% of patients on active treatment with the drug, a relatively uncommon situation that remains to be explained [12,18]. While there is no consensus on this matter, based on our clinical experience, we recommend following up on patients who discontinue denosumab with imaging tests at 4-month intervals during the first two years, in order to closely monitor for recurrence.

### 4.5. Toxicity

Approximately one-third of our patients experienced an adverse event over the course of their treatment, albeit most of these were of little clinical significance. Regardless of the therapeutic strategy employed, denosumab tends to be well tolerated. However, a watchful eye should be kept on patients to detect severe adverse events such as osteonecrosis of the jaw or atypical bone fractures, particularly on those receiving the drug over long periods of time, as toxicity is closely related to the duration of administration of the drug. Our findings regarding these major complications support this observation, as an atypical femoral fracture presented in a patient who had received denosumab as part of their treatment strategy for more than two years, and the median time to onset for jaw osteonecrosis was four years. For this reason, several authors have advocated the implementation of drug holidays or dose reductions in patients treated over the long term in order to balance the need to minimize side effects and keep the tumor in check at the same time [18,22,38].

### 4.6. Limitations of This Study

This study is not without limitations. Firstly, it is a retrospective analysis including heterogeneous cases in terms of location and severity. Its retrospective design and the participation of seven different hospitals limited our ability to collect certain variables of interest, such as tumor size or pain quantitative scales. However, we believe that our analysis accurately reflects the clinical reality and current management of GCTBs in Spain. Moreover, the mentioned variables were indirectly captured through other recorded parameters, such as radiological and clinical response. Secondly, we did not systematically apply a standardized staging system used in malignant tumors, such as the TNM classification. While we acknowledge that this could offer valuable oncologic insight, Campanacci’s radiological classification appears to be the most widely used system for staging GCTBs, which is why we included it in our study. Lastly, the small and diverse nature of our patient series limited our ability to perform the more robust statistical analyses we had intended, and some of the analyses we did conduct may have been underpowered due to the sample size. As a result of these limitations, a prospective analysis should be conducted of the three treatment strategies reviewed here in order to establish comprehensive guidelines that facilitate decision-making within multidisciplinary tumor boards.

## 5. Conclusions

Neoadjuvant patients treated with curettage who experienced local recurrence had received denosumab for longer periods, although no statistically significant differences were found. Reintroduction of denosumab in the neoadjuvant group did not prevent recurrence. Recurrence rates did not differ significantly between neoadjuvant and adjuvant strategies. Discontinuation of single treatment with denosumab did not necessarily lead to progression of GCTBs.

## Figures and Tables

**Table 1:** Patient anthropometric and clinical characteristics.

			Neoadjuvant Treatment	Adjuvant Treatment	Single Treatment
Sex					
	Male		12 (31.6%)	1 (12.5%)	12 (44.4%)
	Female		26 (68.4%)	7 (87.5%)	19 (70.4%)
Age (years)			38.0 ± 13.7 [16–64]	26.0 ± 7.2 [15–39]	39.5 ± 13.4 [18–63]
Origin					
	Primary		32 (84.2%)	7 (87.5%)	16 (59.3%)
	Recurrence		6 (15.8%)	1 (12.5%)	11 (40.7%)
Location of the tumor					
	Axial skeleton		-	1 (12.5%)	5 (18.5%)
	Proximal humerus		-	1 (12.5%)	-
	Distal humerus		1 (2.6%)	-	-
	Distal radius		6 (15.8%)	-	3 (11.1%)
	Distal ulna		1 (2.6%)	2 (25.0%)	-
	Pelvis		-	1 (12.5%)	11 (40.7%)
	Distal femur		14 (36.8%)	-	2 (7.4%)
	Proximal femur		2 (5.3%)	-	-
	Proximal tibia		7 (18.4%)	1 (12.5%)	3 (11.1%)
	Distal tibia		1 (2.6%)	-	-
	Hand				
		Phalanges	3 (7.9%)	-	-
	Foot				
		Talus	1 (2.6%)	2 (25.0%)	2 (7.4%)
		Cuneiforms	-	-	1 (3.7%)
		Scaphoids	1 (2.6%)	-	-
Campanacci’s radiological classification					
	I		-	-	1 (3.7%)
	II		17 (44.7%)	1 (12.5%)	14 (51.9%)
	II with fracture		3 (7.9%)	2 (25.0%)	2 (7.4%)
	III		15 (39.5%)	5 (62.5%)	10 (37.0%)
	Unknown		3 (7.9%)	-	-

**Table 2:** Median duration of denosumab administration (months) by treatment strategy. For patients receiving neoadjuvant treatment followed by surgery, the median duration of reintroduction therapy includes only those who had completed the treatment at the end of this study.

Treatment Strategy		Median	Range
Neoadjuvant treatment		6.1	0.5–19.8
	Reintroduction after curettage	4.8	0.9–7.5
Adjuvant treatment			
	Off-denosumab	15.3	3.1–19.3
	On-denosumab	12.8	0.4–78.3
Single treatment			
	Off-denosumab	34.2	5.0–83.2
	On-denosumab	51.8	14.6–130.7

**Table 3:** Adverse events related to denosumab administration stratified by treatment strategy.

	Neoadjuvant Treatment	Adjuvant Treatment	Single Treatment
Jaw osteonecrosis	-	-	5 (18.5%)
Pain in the limbs	8 (21.1%)	1 (12.5%)	5 (18.5%)
Backache	3 (7.9%)	-	1 (3.7%)
Muscle weakness	7 (18.4%)	1 (12.5%)	3 (11.1%)
Diarrhea	-	-	1 (3.7%)
Nausea	3 (7.9%)	-	-
Fatigue	6 (15.8%)	3 (37.5%)	4 (14.8%)
Headache	2 (5.3%)	1 (12.5%)	1 (3.7%)
Hypocalcemia	1 (2.6%)	-	-
Respiratory failure	1 (2.6%)	-	-
Atypical fracture	1 (2.6%)	-	-
Leg cramps	-	-	1 (3.7%)
Myalgia	-	-	1 (3.7%)
Anemia	-	-	1 (3.7%)
Hypertransaminasemia	-	-	1 (3.7%)
Dizziness	1 (2.6%)	-	-
Asthenia	1 (2.6%)	-	1 (3.7%)
Neutropenia	-	-	1 (3.7%)
Insomnia	-	-	1 (3.7%)

## Data Availability

The datasets used and/or analyzed during the current study are available from the corresponding author on reasonable request.

## Abbreviations

The following abbreviations are used in this manuscript:

AEMPS	Spanish Medicines Agency
CTCAE	Common Terminology Criteria for Adverse Events
GCTB	Giant-cell tumors of the bone
ICDS	Inverse Choi Density/Size
LIETAL	Spanish Musculoskeletal Tumors Research Consortium
RANKL	Receptor activator of nuclear factor kappa-B ligand
SECOT	Spanish Society of Orthopedics and Trauma Surgery
